# Congenital diaphragmatic Bochdaleck hernia: case report

**DOI:** 10.1186/1755-7682-5-30

**Published:** 2012-10-30

**Authors:** Jamile Rizzardi Lava, Guilherme A Hettwer, Cleiton Jonei Reginatto, Guilherme Galoro, Carolina T Gehlen, Maria CM Subtil, Vitor E Valenti, Luiz Carlos deAbreu, Carlos Bandeira de Mello Monteiro, Márcio Petenusso

**Affiliations:** 1 Departamento de Medicina, Universidade do Planalto Catarinense, Avenida Castelo Branco, Lages, SC, 88509-900, Brazil; 2 Departamento de Fonoaudiologia, Faculdade de Filosofia e Ciências, Universidade Estadual Paulista, UNESP, Av. Hygino Muzzi Filho, 646, Marília, SP, 17525-900, Brazil; 3 Laboratório de Escrita Científica, Departamento de Morfologia e Fisiologia, Faculdade de Medicina do ABC, Av. Príncipe de Gales, 821, Santo André, SP, Brazil

**Keywords:** Hernia, Diaphragmatic, Prenatal Care, Infant, Newborn

## Abstract

Congenital diaphragmatic Bochdaleck hernia is an anatomical defect of the diaphragm, which allows protrusion of abdominal viscera into the chest, causing serious pulmonary and cardiac complications in the neonate. In this study we aimed to present a case of congenital Bochdaleck hernia. We investigated a 40 weeks old child, with a pregnancy carried out in a public hospital in Passo Fundo, Rio Grande do Sul, Brazil. We suggest that if diagnosis occurs in the prenatal period, the prognosis of this disease improves. As a consequence, it allows the parity of the fetus to occur in a higher complexity center, optimizing the chances of survival.

## Background

The diaphragm is a modified half-dome of musculofibrous tissue that separates the thorax from the abdomen [1-3]. Diaphragmatic hernia is a general term used to indicate protrusion of abdominal viscera into the chest cavity through communication, which is congenital or acquired [4]. The congenital diaphragmatic hernia (CDH) is classified according to the location of the protrusion, including in hiatal hernia, Morgagni-Larrey hernia and Bochdaleck hernia [5].

The Bochdaleck hernias are the result of inadequate obliteration of the lumbar elements in the pleuroperitoneal area, during the eighth and tenth week of intrauterine development [6]. This pathological condition was first described by Bochdaleck in 1848 and it is usually presents in childhood with an incidence ranging from 1:4000 to 1:7000 newborns [7]. It is rarely seen in adults, with little more than 100 cases reported in the literature [8].

The literature focused on the identification of factors useful for predicting poor outcome in fetuses and neonates with CDH. Previously, some researchers suggested the merit of pulmonary artery–based measurements, whereas the predictive value of observed-to-expected lung-to-head ratio was extensively discussed during the past decade [9,10]. In contrast, liver position has gained wide acceptance as an excellent prognostic factor and is considered by many to be the best prenatal predictor of outcome [11,12].

When diagnosed in a newborn, congenital diaphragmatic hernias are frequently associated with significant respiratory distress and mortality [13]. Also, delay in the diagnosis of diaphragmatic hernias may result in increased morbidity. Intestinal strangulation in late-presenting, left Bochdalek congenital hernia was reported in previous investigations [14,15]. Taken together, our case report and the literature highlight the importance of obtaining and accurately interpreting a chest radiograph in children with unexplained respiratory or gastrointestinal symptoms. Moreover, once the diagnoses were made, repair should be undertaken expeditiously, before complications development.

In 85-90% of cases the protrusion occurs in the left hemithorax, probably by early closure of the right pleuroperitoneal opening [16]. Moreover, the CDH is the most common diaphragmatic hernia, occurring in 90% of cases and is associated with a high neonatal mortality ranging from 40 to 80% of cases [17]. In this study we reported a case of CDH in a child.

## Case report

We investigated a male patient, born at term by vaginal delivery with difficult extraction, weighing 3575 g, with Apgar score 3/7 in the first and fifth minutes of life, which required positive pressure ventilation (PPV) at birth. The parents signed a consent letter that allowed the authors to publish this case report. His mother did not make complete prenatal care and has no ultrasonographic examination of the current pregnancy.

The newborn was born well and evolved with sudden respiratory distress, requiring ventilation. The newborn should be examined for murmurs, to be conducted as a congenital and/or abdomen dug present for diaphragmatic hernia, being intubated immediately once he presents any clinical diagnostic. Approximately twenty minutes after birth, the newborn had a sudden respiratory dysfunction evolved into a prolonged cardiac arrest. The newborn was intubated and received cardiac massage and resuscitation. He was then taken to the Intensive Care Unit and carried out a chest radiograph, which showed atelectasis of the right lung and left diaphragmatic hernia. He developed acute renal failure and was not in condition to perform peritoneal dialysis, as well as surgical correction of the hernia. He died in the second day of life.

Blood tests were collected during ICU admission, however, it was not ready on time. The patient died before we perform analysis. We also performed other examinations such as examination without contrast and Simple acute abdomen X-ray. Echocardiography was done by initial suspicion of congenital heart disease, discarding the diagnostic. We found no clinical relevant findings related to cardiac dysfunction, since the values regarding ejection fraction, diastolic and systolic function was considered in the physiological standards. Thus, it was requested the acute abdomen X-ray.

**Figure 1:**
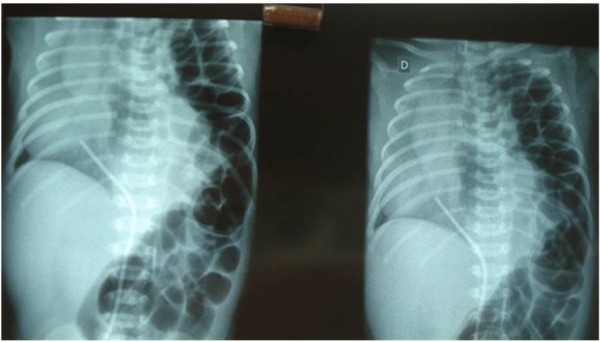
X-ray indicating diaphragmatic Bochdaleck.

According to Figure 1, we observe small bowel in the chest through a posterolateral hole, shifting the mediastinum to the right. It is also possible to observe in this Figure the presence of a tube on the right lower hemithorax when the newborn was at supine position, this tube was necessary to remove pus from the intrathoracic space due to the inflammation mechanism in the area.

## Discussion

Through the diaphragmatic defect of the Bochdaleck hernia, abdominal organs (intestines, spleen, stomach, kidney) may migrate into the pleural cavity, compressing the lung and displacing the mediastinum to the opposite side [18]. Depending on the degree of pulmonary compression, there may be a marked decrease of the pulmonary branches, limited multiplication of alveoli, and muscle hypertrophy in pulmonary arterioles, leading to a decreased functional lung mass (pulmonary hypoplasia), involving both lungs with the ipsilateral lung more affected. These newborns are anatomically predisposed to develop Persistent Pulmonary Hypertension, maintaining the permanence of the fetal circulation to the lungs with blood moved through the foramen ovale and ductus arteriosus. The right-left shunt causes acidosis and hypoxia, increased pulmonary vasoconstriction and worsening pulmonary hypertension. It may lead to decreased lung compliance [19].

The clinical profile of respiratory distress reported in our case may progress to severe respiratory distress and lung failure. During physical examination it may be seen excavated abdomen, displaced heart sounds, respiratory failure and bowel sounds in the chest. During the radiography we noted a hemithorax filled cyst-like structures (bowel), mediastinum and abdomen relatively free of gas. We should mention the importance of the differential diagnosis with cystic adenomatoid malformation of the lung and pneumonia [20].

Treatment in severe cases and prenatal diagnosis is recommended an immediate oxygenation. When it is observed an inadequate prenatal, the morphologic ultrasound examination is the best used during prenatal period. Moreover, once the diaphragmatic hernia diagnostic is done, the newborn should be immediately intubated, in order to perform immediate decompression of the chest with abdominal straps, preventing pulmonary hypertension. All newborns should be immediately intubated after birth or at diagnosis, with the introduction of a nasogastric tube on continuous suction. We should be careful with assisted ventilation in order to maintain a low inspiratory pressure, to avoid damage or breakage of the contralateral lung. Surgical repair should be performed only after stabilization, through subcostal laparotomy with reduction of hernial contents into the abdominal cavity and closure of the diaphragmatic defect. The binomial hypoplasia, pulmonary hypertension remain a major mortality factor [21].

According to our case report, we believe that it is helpful in terms of providing neonatologists, surgeons and maternal-fetal medicine specialists with realistic prognostic information for counseling families. Nonetheless, several cautions are worth noting. Furthermore, it was recently established a multidisciplinary CDH clinic that was composed by specialists from a variety of areas, including pediatric general surgery, pulmonary, cardiology and nutrition [22]. This clinic allows us to make a more in-depth evaluation of the CDH and it certainly allows for better follow-up of children with this disease in a prospective mode.

Our study presents a point that should be addressed, although the newborn underwent invasive ventilation, our tool was not able to process the data regarding blood gas analysis. It is because the time was not enough for the results to be read due to the premature death of the newborn, since the premature newborn did not survived two entire days.

## Conclusion

The diagnosis in the pre-natal period induces to a better prognosis, since the birth in a center well equipped will made necessary interventions, minimizing neonatal complications and, thus, optimizing the chances of survival. The clinical importance overlaps the X ray in case of no diagnosis with prenatal care, it is very important for physicians to be alert for signs that show a possible case of diaphragmatic hernia.

## Competing interests

We declare no conflict of interest.

## Authors’ contributions

JL, GAH, CR, CR, GG, CTG, MCMS, VEV, LCdeA, CBdeMM and MP participated in the acquisition of data and revision of the manuscript. All authors conceived of the study, determined the design, interpreted the data and drafted the manuscript. VEV and LCA determined the design and drafted the manuscript. All authors read and gave final approval for the version submitted for publication.

## References

[B1] Kaya B Bat O Esenbulut N Memisoglu K The intraabdominal bleeding with an inguinal defect that mimicking a femoral vein aneurysm. A case report Int Arch Med 2011 4 13 13 10.1186/1755-7682-4-13 21496325 PMC3090334

[B2] Piciucchi S Milandri C Verdecchia GM Framarini M Amadori E Monti M Oboldi D Bandi G Barone D Gavelli G Acute hiatal hernia: a late complication following gastrectomy Int Arch Med 2010 3 23 23 10.1186/1755-7682-3-23 20920326 PMC2958858

[B3] Sodhi SS Zech LA Jr Batura V Kulasekhar S Diaphragmatic hernia with strangulated loop of bowel presenting after colonoscopy: case report Int Arch Med 2009 2 38 38 10.1186/1755-7682-2-38 20003353 PMC2797493

[B4] Rey L Dicionário de termos técnicos de medicina e saúde 2003 2 Editora Guanabara Koogan S/a, Rio de Janeiro 950

[B5] Mandhan P Memon A Memon AS Congenital herniasofthediaphragm in children. JournalofAyub Medical College, Abbottabad JAMC 2007 19 37 41 18183717

[B6] Chiu PP Langer JC Surgicalconditionsofthediaphragm: posterior diaphragmatichernias in infants Thor Surger Clinic 2009 19 451 61 10.1016/j.thorsurg.2009.08.009 20112627

[B7] Loch LF Hérnia diafragmática congênita de apresentação tardia Rev AMRIGS 2008 52 212 215

[B8] Ruano R Prenataldiagnosisand perinatal outcomeof 38 cases with congenital diaphragmatichernia: 8-year experienceof a tertiaryBraziliancenter Clinics 2006 61 122 127

[B9] Yang SH Nobuhara KK Keller RL Ball RH Goldstein RB Feldstein VA Callen PW Filly RA Farmer DL Harrison MR Lee H Reliability of the lung-to-head ratio as a predictor of outcome in fetuses with isolated left congenital diaphragmatic hernia at gestation outside 24–26 weeks Am J Obstet Gynecol 2007 197 30.e1-7 17618746 10.1016/j.ajog.2007.01.016

[B10] Jani J Nicolaides KH Keller RL Benachi A Peralta CF Favre R Moreno O Tibboel D Lipitz S Eggink A Vaast P Allegaert K Harrison M Deprest J Antenatal-CDH-Registry Group Observed to expected lung area to head circumference ratio in the prediction of survival in fetuses with isolated diaphragmatic hernia Ultrasound Obstet Gynecol 2007 30 67 71 10.1002/uog.4052 17587219

[B11] Hedrick HL Danzer E Merchant A Bebbington MW Zhao H Flake AW Johnson MP Liechty KW Howell LJ Wilson RD Adzick NS Liver position and lung-to-head ratio for prediction of extracorporeal membrane oxygenation and survival in isolated left congenital diaphragmatic hernia Am J Obstet Gynecol 2007 197 422.e1-4 17904987 10.1016/j.ajog.2007.07.001

[B12] Kitano Y Nakagawa S Kuroda T Honna T Itoh Y Nakamura T Morikawa N Shimizu N Kashima K Hayashi S Sago H Liver position in fetal congenital diaphragmatic hernia retains a prognostic value in the era of lung-protective strategy J Pediatr Surg 2005 40 1827 32 10.1016/j.jpedsurg.2005.08.020 16338299

[B13] van den Hout L Reiss I Felix JF Risk factors for chronic lung disease and mortality in newborns with congenital diaphragmatic hernia Neonatology 2010 98 370 380 10.1159/000316974 21042035

[B14] Elhalaby EA Abo Sikeem MH Delayed presentation of congenital diaphragmatic hernia Pediatr Surg Int 2002 18 480 485 10.1007/s00383-002-0743-1 12415386

[B15] Weber TR Tracy T Bailey PV Congenital diaphragmatic hernia beyond infancy Am J Surg 2002 162 643 646 1670242 10.1016/0002-9610(91)90127-y

[B16] Clugston RD Greer JJ Diaphragmdevelopmentand congenital diaphragmatichernia Seminars in Pediat Surg 2007 16 94 100 10.1053/j.sempedsurg.2007.01.004 17462561

[B17] Lazar DA Impactofprenatalevaluationandprotocol-based perinatal management on congenital diaphragmaticherniaoutcomes J Pediat Surg 2011 46 808 813 10.1016/j.jpedsurg.2011.02.009 21616231

[B18] Keijzer R Puri P Congenital diaphragmatichernia Seminars Pediat Surg 2010 19 180 185 10.1053/j.sempedsurg.2010.03.001 20610190

[B19] Leitzke L Diagnóstico Pré-Natal de Hérnia Diafragmática Congênita por imagem de Ressonância Magnética Arqu Catar Medic 2007 36 110 118

[B20] Torre MB Hérnias Diafragmáticas Pediatria 1994 16 133 134

[B21] Wu C Late-presenting congenital diaphragmatichernia in pediatricemergencyroom: two case reports Europ J Pediatr 2009 168 1013 1015 10.1007/s00431-008-0874-z 19011899

[B22] Gray BW Fifer CG Hirsch JC Tochman SW Drongowski RA Mychaliska GB Kunisaki SM Contemporary Outcomes in Infants With Congenital Heart Disease and Bochdalek Diaphragmatic Hernia Ann Thorac Surg 2012 Epub ahead of print 10.1016/j.athoracsur.2012.07.010 22939449

